# Evaluation of Salivary Indoxyl Sulfate with Proteinuria for Predicting Graft Deterioration in Kidney Transplant Recipients

**DOI:** 10.3390/toxins13080571

**Published:** 2021-08-16

**Authors:** Natalia Korytowska, Aleksandra Wyczałkowska-Tomasik, Leszek Pączek, Joanna Giebułtowicz

**Affiliations:** 1 Department of Bioanalysis and Drugs Analysis, Faculty of Pharmacy, Medical University of Warsaw, 1 Banacha, 02-097 Warsaw, Poland; natalia.korytowska@wum.edu.pl; 2 Department of Immunology, Transplantology, and Internal Diseases, Medical University of Warsaw, 59 Nowogrodzka, 02-006 Warsaw, Poland; atomasik@wum.edu.pl (A.W.-T.); leszek.paczek@wum.edu.pl (L.P.)

**Keywords:** chronic kidney disease, diagnostic biomarkers, indoxyl sulfate, LC-MS, proteinuria, renal replacement therapy, saliva, urine, uremic toxicity

## Abstract

Acute kidney injury (AKI) is a significant risk factor for developing chronic kidney disease and progression to end-stage renal disease in elderly patients. AKI is also a relatively common complication after kidney transplantation (KTx) associated with graft failure. Since the lifespan of a transplanted kidney is limited, the risk of the loss/deterioration of graft function (DoGF) should be estimated to apply the preventive treatment. The collection of saliva and urine is more convenient than collecting blood and can be performed at home. The study aimed to verify whether non-invasive biomarkers, determined in saliva and urine, may be useful in the prediction of DoGF in kidney transplant recipients (KTRs) (*n* = 92). Salivary and serum toxins (*p*-cresol sulfate, pCS; indoxyl sulfate, IS) concentrations were determined using liquid chromatography-tandem mass spectrometry (LC-MS/MS). Urinary proteins, hemoglobin, and glucose were measured using a semi-quantitative strip test. Salivary IS (odds ratio (OR) = 1.19), and proteinuria (OR = 3.69) were demonstrated as independent factors for the prediction of DoGF. Satisfactory discriminatory power (area under the receiver operating characteristic curve (AUC) = 0.71 ± 0.07) and calibration of the model were obtained. The model showed that categories of the increasing probability of the risk of DoGF are associated with the decreased risk of graft survival. The non-invasive diagnostic biomarkers are a useful screening tool to identify high-risk patients for DoGF.

## 1. Introduction

Acute kidney injury (AKI) is a significant risk factor for developing chronic kidney disease (CKD) and progression to end-stage renal disease (ESRD) in elderly patients [1]. Both dialysis and kidney transplantation (KTx) are treatments for patients with ESRD [2]. Dialysis is a life-extending treatment that removes waste, toxins, and excess fluid [3,4]. However, dialysis-related complications such as muscle cramps, itching skin, dizziness, headaches, and fatigue or general lethargy significantly decrease the quality of life of patients [2,5]. Thus, KTx is a preferred type of renal replacement therapy [6]. It reduces cardiovascular risk and mortality, although the risk remains higher than in the general population [7]. However, the functioning of the transplanted kidney is time-limited. Allograft survival is influenced by factors like the pre-transplant profile of the donor and recipient. These include donor age and weight and recipient body mass index (BMI) and sex [8], factors connected with surgery such as length of cold ischemia time [9], as well as with post-transplant factors, e.g., immunosuppressive therapy [10]. AKI is also a relatively common complication after KTx associated with graft failure [1]. The decreasing function of the kidney might require a return to dialysis or retransplantation. Thus, the identifying of predictors for loss or deterioration of graft function to extend its satisfactory functioning is a crucial issue for both clinicians and kidney transplant recipients (KTRs) [11,12].

The prediction and prevention of loss or deterioration of graft function are essential for patients and the effectiveness of medical care. Long waiting times for renal transplantation results in dialysis have lasted up to 11 years [11]. Unfortunately, the dialysis time longer than 1 year is a poor predictor for graft survival [13]. Another poor predictor is the age of the donor. Nevertheless, the aged donors are accepted to minimize the waiting time and reduce the risk of early death [14,15]. Various factors have been explored that influence short- and long-term graft loss. Risk prediction models for graft failure after KTx, reported in all of the 31 studies reviewed by Kabore et al. [12] required invasive blood tests or complex data regarding the donor and recipient such as cold ischemic time, HLA mismatch, or comorbidities. Non-invasive urinary tests such as monocyte chemoattractant protein 1 (CCL2), albumin-creatinine ratio or proteinuria, are useful in a prediction of graft loss, but only as a part of the model with blood tests and/or the use of clinical data [12]. Up to now, the use of saliva as the non-invasive material to this aim was not reported. Saliva has already been proven as a valuable clinical material to assess kidney function. Until now, not only well-known biomarkers such as creatinine [16] but also new markers such as uremic toxins [17] have been recognized as a reliable salivary marker of kidney function.

This study aimed to assess the utility of variables tested in non-invasively collected materials (saliva and urine) for predicting the deterioration of graft function in KTRs beyond 1-year KTx. The pre-transplant data are suggested to influence only short-term survival, whereas long-term survival can be predicted using laboratory tests and other parameters determined with the use of post-transplant data. Therefore, we used only the data obtained at the time of the patient enrolment. In the previous study, we showed a correlation between serum and salivary concentrations of pCS and IS (r = 0.74 and r = 0.81, respectively) in chronic kidney disease (CKD) patients [17]. In this work, the usefulness of saliva to estimate pCS and IS concentration in the blood of KTRs was first examined. Additionally, the utility of such variables in urine was tested including protein, hemoglobin, and glucose level determination. Patients were also characterized by standard clinical and blood parameters. Furthermore, eGFR was used as a marker of kidney function to estimate the correlation with toxins and was not included in the model since it required a blood analysis (invasively collected material). The blood, saliva, and urine were collected three times a day at six monthly intervals.

## 2. Results

### 2.1. Baseline Patients’ Characteristics

The baseline study group included 37 Caucasian females and 55 Caucasian males, with the median (M) age of 53 (IQR = 19) years. The time after kidney transplant ranged from 279 to 1828 days (M = 1125 days, IQR = 844 days). The clinical blood, urinary, and salivary parameters for the study population of the 92 kidney recipients at M1, M6 (*n* = 77), and M12 (*n* = 73) are shown in Table 1.

The most common immunosuppressive drug regimen combined corticosteroids, a calcineurin inhibitor, and an anti-proliferative drug. The most common cause of ESRD was glomerulonephritis (40%), diabetic nephropathy (10%), and polycystic kidney disease (28%). Other reasons included another type of nephritis, hypertensive kidney disease, Alport syndrome, and kidney cancer. For most of the patients, it was a primary (87%) or secondary transplant (12%) from a deceased donor (90%). For one patient it was the third transplantation. The duration of follow-up ranged from 354 to 1958 days (M = 1706 days, IQR = 390 days), from baseline (M1) to about 41 (12) months after the last collection.

### 2.2. Evaluation of Graft Function—eGFR, Salivary and Serum pCS and IS

The median concentration of IS and pCS in serum and saliva, as well as for eGFR, during study follow-up, was calculated (Table 1). There were no differences between concentrations of toxins in both diagnostic materials between baseline (M1) and the next collections (M6, M12). Significant differences were observed between eGFR values in M6 and M12 (*p* = 0.00807).

**Figure 1:**
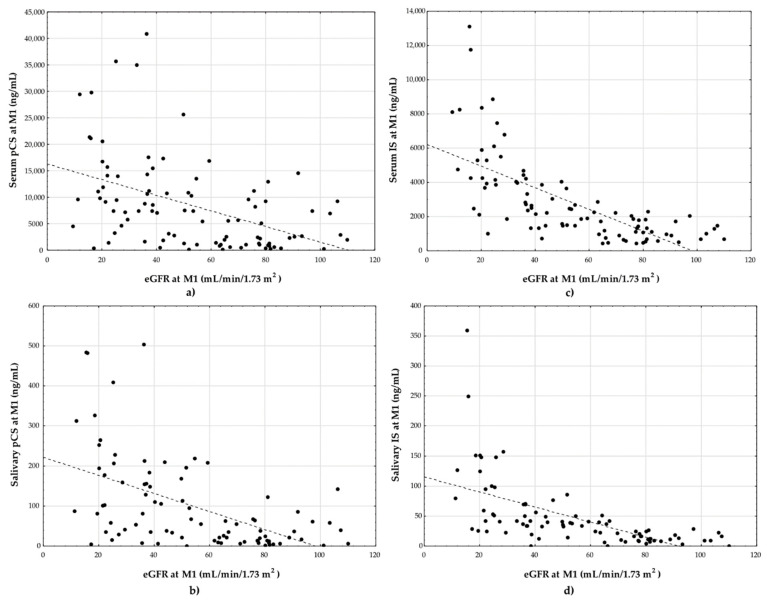
The correlation between eGFR and (**a**) serum pCS (r_s_ = −0.50); (**b**) salivary pCS (r_s_ = −0.55); (**c**) serum IS (r_s_ = −0.78); and (**d**) salivary IS (r_s_ = −0.76) at M1 for all subjects. eGFR: estimated glomerular filtration rate; IS: indoxyl sulfate; pCS: *p*-cresol sulfate.

There was a significant correlation at M1, between both toxins in serum and saliva with eGFR for all subjects (Figure 1), for the DoGF-free group and the DoGF group (Table 2). The only correlation between salivary pCS and eGFR in the DoGF group was insignificant.

**Figure 2:**
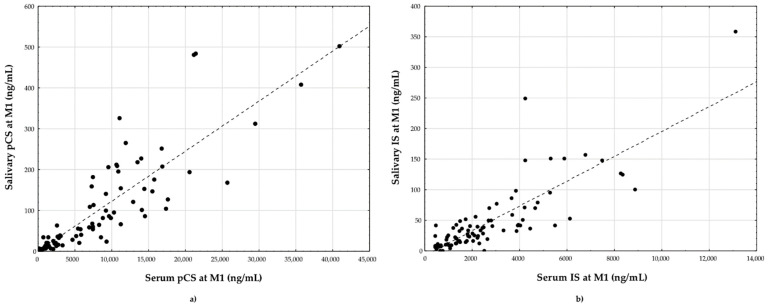
The correlation between (**a**) salivary and serum pCS (r_s_ = 0.92); (**b**) salivary and serum IS (r_s_ = 0.81) at M1 for all subjects. IS: indoxyl sulfate; pCS: *p*-cresol sulfate.

Moreover, high significant correlations were observed between salivary and serum IS at M1 (r_s_ = 0.81, *p* < 0.00001), and between salivary and serum pCS at M1 (r_s_ = 0.92, *p* < 0.00001) (Figure 2).

### 2.3. Analysis of Risk Factors for Graft Deterioration

To identify salivary and urinary biomarkers associated with the deterioration of graft function the univariate regression analysis was applied (Table 3). The deterioration of graft function was defined as progression to ESRD (3 out of 92), progressive ESRD (2 out of 92), the decline of eGFR above 40% (6 out of 92), the return to chronic dialysis (7 out of 92) or death due to urosepsis (2 out of 92; one patient with diagnosed rejection process) during follow-up. Lack of contact with patients (1 out of 92) was an exclusion criterion from the final calculations. Two variables were found to be associated with the deterioration of graft function: salivary IS (10 ng/mL) and the presence of proteinuria. Salivary pCS, presence of hematuria, and presence of glucosuria were not risk factors for the deterioration of graft function. The influence of change of concentration, both pCS and IS in saliva, during the observation period were also analyzed; however, the univariate logistic analysis showed that analyzed variables were statistically insignificant (*p*-value for % of difference between M1 and M6 for salivary pCS and IS was equal to 0.2252 and 0.7205, accordingly; *p*-value for % of difference between M1 and M12 for salivary pCS and IS was equal to 0.8480 and 0.7063, accordingly).

All variables statistically significant in the univariate analysis (*p* < 0.05) were included in the multivariate logistic regression analysis. The most suitable variables for our model were selected using a backward elimination algorithm. According to the algorithm used, salivary IS and proteinuria were identified as independent predictors of deterioration of graft function (Table 3).

**Figure 3:**
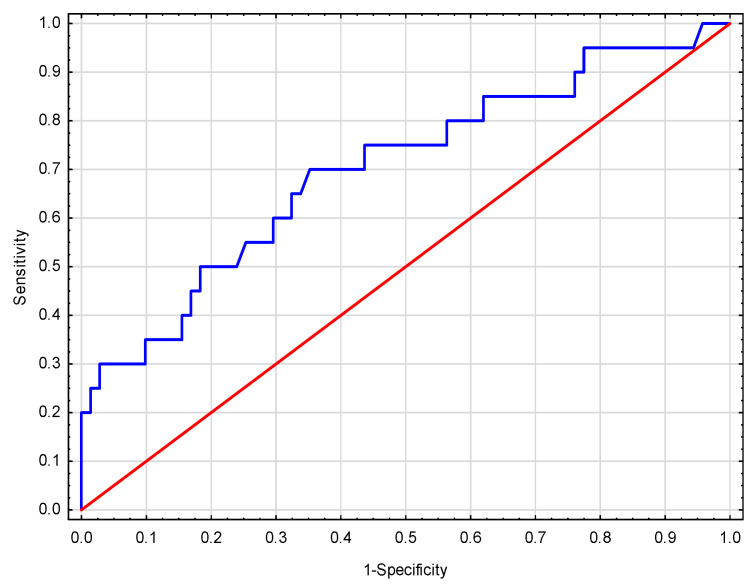
The area under the receiver operating characteristic curve (AUC) for the model of deterioration of graft function included salivary IS and proteinuria at M1.

The Hosmer–Lemeshow goodness-of-fit test showed that the model received adequate calibration (χ^2^ = 7.3441, *p* = 0.5000) [19]. The acceptable predictive value of the model (area under the receiver operating characteristic curve (AUC): 0.710 ± 0.07) was demonstrated to identify patients with a higher risk of deterioration of graft function (Figure 3).

### 2.4. Predicted Probability of Deterioration of Graft Function

**Figure 4:**
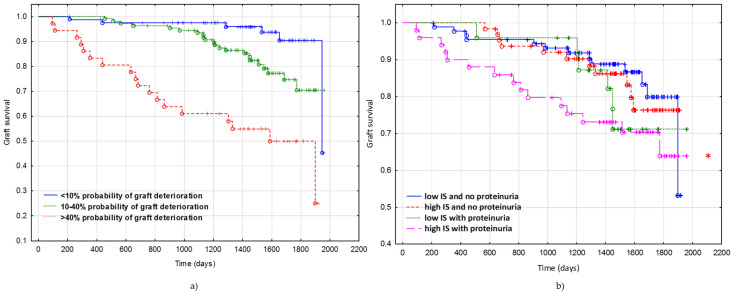
Graft survival according to the (**a**) categories of predicted probabilities of deterioration of graft function: calculated risk of deterioration of graft function lower than 10%, between 10% and 40%, and above 40% (*p* < 0.00001); (**b**) sub-groups: low IS and no proteinuria, high IS and no proteinuria, low IS with proteinuria, and high IS with proteinuria. * The only statistically significant difference was observed between the group with low IS and no proteinuria and the group with high IS with proteinuria (*p* = 0.04250).

A Kaplan–Meier analysis was performed to investigate the association between the predicted probabilities of deterioration of graft function as calculated from the received multivariable logistic model and graft failure in our cohort. The cut-off values for predicted probabilities to group the patients were chosen as: <10% risk of deterioration of graft function, 10–40% risk, and >40% risk. The log-rank test showed a significant association between high predicted probabilities of deterioration of graft function and deterioration of graft function (*p* < 0.00001) (Figure 4a). Since high salivary IS and the presence of proteinuria may be risk factors of DoGF, the Kaplan–Meier analysis divided the subjects into sub-groups: low IS and no proteinuria, high IS and no proteinuria, low IS with proteinuria, and high IS with proteinuria (Figure 4b).

## 3. Discussion

We have demonstrated that monitoring of kidney functioning is feasible using non-invasive tests and easily available diagnostic material, i.e., saliva and urine. We have shown that salivary IS (OR = 1.19) and proteinuria (OR = 3.69) are the independent factors for long-term predicting of the deterioration of graft function in KTRs, beyond 1 year of kidney transplantation. Proteinuria showed the strongest correlation with the deterioration of graft function (OR = 3.69). Mottola C. et al. also showed a strong correlation between proteinuria and poor graft survival but measured only within 1 year of Ktx [20]. Proteinuria is also a risk factor for death in the first post-transplant year [10] and cardiovascular diseases in KTRs [21]. Moreover, there is the association between proteinuria and the recurrence of primary glomerulonephritis [22,23]. Hematuria (*p* = 0.07142) and glucosuria (*p* = 0.05781) are not risk factors for the deterioration of graft function. When other end-points were established (progression to ESRD, progressive ESRD, return to chronic dialysis, or death due to urosepsis) hematuria was included in the model (data not published). However, due to a small number of events per variable, that model was not applied. Hematuria frequently occurs following Ktx, mostly due to urologic malignancy and urinary tract infection [24,25]. Hematuria has been already reported as an independent risk factor for CKD progression and death [26], but not for KTRs. Hematuria was also connected with a higher risk of ESRD in patients aged 16–25 years for 22 years [27].

Contrary to the salivary IS, salivary pCS was not useful to predict the deterioration of graft function. Lack of correlation between salivary pCS and the deterioration of graft function might indicate that pCS was involved in the pathogenesis of another disease in KTRs. For instance, pCS is associated with a cardiovascular event in patients with CKD [28] and diabetes in elderly dialysis patients [29].

The calibration and discriminating power for the deterioration of graft function of our preliminary prediction model were assessed. Firstly, the discrimination was quantified with an ROC analysis. This analysis confirmed the good discriminatory power and identified the presence of the deterioration of graft function, with an AUC of 0.71 ± 0.07. A systematic review including 31 studies on risk prediction models for graft failure after KTx showed that the discriminatory power of presented models ranged from 0.55–0.90 [12]. Secondly, the Hosmer–Lemeshow “goodness-of-fit” test was used to quantify calibration, where the good capacity of the model to estimate correctly the probability of the deterioration of graft function was shown. Lastly, we have used our model to show that increasing categories of deterioration of graft function risk probability (<10%, 10–40%, and >40% risk of deterioration of graft function) are associated with a decreased risk of graft survival.

Compared to the existing solutions, our model is very straightforward and requires the use of only two variables. Some other models for long-term predictions (>1 year of follow-up) of graft loss obtained satisfactory results using complex recipients’ tests and including as many as 48 [30] or 38 clinical variables [31]. These models also used data on donors including age, hepatitis C virus status, types, and creatinine level [32]. Other models also required additional laboratory tests (e.g., recipient blood creatinine) measured at specified time points after KTx, i.e., 3 and 12 months [33]. Our model is a unique one requiring only a non-invasive, simple, two variables test to predict long-term loss/deterioration of graft function for patients who survived the first post-transplant year.

Our model includes variables collected only during patients’ enrolments at different times after KTx. Models for long-term follow-up are usually very heterogeneous [12]. Some of them include only parameters determined at the time of transplantation like the age of the donor and recipient or a number of mismatches in haplotype between the donor and recipient [34]. Others also include predictors determined from 1 week [30] to 1 year [33] after transplantation. For the practical use of the model as a predictive tool it is important to plan its application. Firstly, a model built with variables measured before or at the time of transplantation [35] can be used for the prediction of graft survival for new patients. Secondly, since the patient’s condition changes during the long-term follow-up, the prediction model can be based on observations after the transplantation [36]. In our study, the second approach was applied. We decided to concentrate only on patients beyond 1 year from surgery. This is due to different causes of graft failure during the first year (surgical complications, early relapses of initial disease), and long-term survival (e.g., immunosuppression) [10,33]. Moreover, within one year from transplantation, the patients’ conditions stabilize at a different rate, which makes the group very heterogeneous. In such a group the statistical analysis is very demanding. Kabore et al. showed that among the 31 studies, 25 papers did not specify inclusion criteria regarding the minimal time after transplantations, one paper selected patients after six, and five papers (similarly as in our study) after twelve months from transplantation [12].

There is a limited number of prognostic models based only on easily collected material. Some models include urinary markers like CCL2 (other parameters: recipient age and delayed graft function) [37], albumin–creatinine ratio (other parameters: recipient age, acute rejection, eGFR, serum albumin) [36], or proteinuria (acute rejection during the first year post-transplant, creatinine at 3 months and creatinine at 12 months) [33], and other factors. In our study, proteinuria was determined by a simple semi-quantitative inexpensive urinary dipstick test (2€ per test). The urinary test strip is simple to use, both in the ward or clinic, as a first-line screening test. The positive results of the test, read visually or using an automated reader, prompt further diagnostics, i.e., quantitative 24 h protein extraction [27,38]. However, all these actions were not needed to build our model. The determination of salivary IS is significantly more expensive (11€ per sample) since it requires the use of liquid chromatography-tandem mass spectrometry (LC-MS/MS).

In our study, only the two uremic toxins were analyzed. IS and pCS are uremic toxins produced by the gut microbiota from dietary tryptophan and tyrosine (or phenylalanine), respectively [39]. Their high levels in the human body are associated not only with CKD progression [17,39] but also with cardiovascular disease and higher mortality in CKD patients [40,41]. The clearance of these toxins by dialysis is limited by strong protein-binding capabilities [42]. Therefore, the monitoring of toxin concentration in KTRs is important due to the poor related prognosis [39,40,41]. Currently, the monitoring of patients after KTx is based mainly on blood and urine examination. We have proven that saliva represents an alternative, non-invasive diagnostic material for the same purpose. In this (r_s_ ≥ 0.81) and our previous paper (r_s_ ≥ 0.70) [17] we proved a strong correlation between serum and salivary toxin levels. Higher correlation in the presented manuscript may be a result of using the currently recommended CKD-EPI equation, while in the previous report the modification of diet in renal disease study equation was applied. However, this is the first study using saliva as the diagnostic material for monitoring the patients after renal transplantation. The use of saliva, as an alternative diagnostic material, offers several advantages over blood. Saliva samples are easy to obtain, even from the elderly and patients having problems with blood donation. The procedure of collecting saliva is easy and can be done by the staff after basic training, and even by patients themselves at home. Therefore, saliva might be considered as the first-line diagnostic sample of choice, especially for monitoring purposes [43].

Reports on the levels of uremic toxins in KTRs are scarce. In this paper, we showed that both IS and pCS are good markers for kidney functions above 1 year after KTx. The correlation coefficient between eGFR and toxins was about two-fold higher for IS than for pCS at each point: M1, M6, and M12. Other studies on the group of KTRs after 5.3 ± 4.9 years from transplantation revealed the association between the stage of CKD and IS, as well as pCS concentration in serum [44]. A similar association was shown for KTRs 8.2 ± 5.7 years after transplantation, where the correlation of pCS with eGFR (r = −0.29) was on a similar level as in our study [45]. On the contrary, no correlation between eGFR and IS was detected for KTRs below 1 year following the renal transplantation [46]. The difference might result from three reasons: (a) differences of excretion of IS and creatinine within 1 year and above 1 year after transplantation (low probability since IS and pCS 24 h urinary excretion were correlated with eGFR [47]); (b) antibiotic therapy, both peri-operative and early after transplantation that changes the structure of gut microbiota and uremic toxins production [48,49]; and (c) other equations used to estimate eGFR, i.e., modification of the diet in renal disease study equation (MDRD) [46] vs. the currently recommended one by KDIGO CKD-EPI equation, which had less bias than the MDRD [18].

In this study, we demonstrated a preliminary model for detecting the deterioration of graft function which can be used as a screening tool to identify high-risk patients for long-term graft loss/deterioration of graft function. Facing limited access to the clinical laboratory, the collection of saliva and urine is much more convenient than collecting blood and can be performed even by patients themselves at home. Moreover, the preparation of the analytical sample from saliva is faster and safer for medical staff. Our preliminary model might be very helpful for the decision-making process to prolong the survival of KTRs, following further studies.

## 4. Conclusions

The convenient and non-invasive model can be useful to predict long-term deterioration of graft function in patients beyond 1 year following KTx. Salivary IS (OR = 1.19), and proteinuria (OR = 3.69) can be considered as independent predictors for the deterioration of graft function (AUC = 0.71 ± 0.07). Our model might be implemented in the clinical decision support system following further studies.

## 5. Materials and Methods

### 5.1. Study Group and Sample Collection

The experimental group included 97 individuals after KTx was randomly selected from patients at the Infant Jesus Teaching Hospital in Warsaw, Poland. The inclusion criterion was renal transplantation conducted between September of 2010 and January of 2015 (from 1 to 5 years before inclusion into this study). Five patients were excluded from the study due to not collecting enough saliva at every time point of the study. Written informed consent was obtained from each subject included in the study and the study design was approved by the Bioethics Committee of the Medical University of Warsaw (No. KB/68/A/2015).

At baseline (M1), all participants completed a questionnaire concerning weight, height, and smoking habits. Smoking status was categorized as: never a smoker, current smoker, former smoker, and passive smoker. BMI was calculated as the ratio of weight (kilograms) to the square of height (meters).

Blood, saliva, and urine collecting were performed at baseline (M1) after 6.0 (interquartile range, IQR = 2.0) months (M6) from M1 and after 7.0 (IQR = 2.0) months (M12) from M6. Additionally, the deterioration of graft function was prospectively collected until November of 2020.

Following the overnight fasting, unstimulated saliva samples were collected using a Salivette device (Sarstedt, Nümbrecht, Germany) with a cotton swab (5 min under the tongue). After collecting, a swab was placed in the Salivette tube and was centrifuged at 750× *g* for 3 min at 4 °C to obtain the saliva samples. Fasting blood was obtained from the cubital vein onto a tube without anticoagulants. The serum was obtained via centrifugation at 340× *g* for 10 min at 20 °C. Then, all samples were stored at −80 °C, until further analysis.

### 5.2. Total Salivary and Serum pCS and IS Form Measurements

Saliva samples were mixed with acetonitrile (1:4, *v*/*v*) and the internal standard to a final concentration of 80 ng/mL. Next, samples were vortexed, incubated at −20 °C for 20 min, and centrifuged at 9300× *g* for 10 min at 4 °C. The supernatant was transferred to a glass vial and analyzed.

Serum samples were mixed with the internal standard (to a final concentration of 600 ng/mL) and methanol (1:1:8, *v*/*v*). Next, samples were incubated at −20 °C for 20 min and centrifuged at 9300× *g* for 10 min at 4 °C. The diluted supernatant (6-fold with mobile phase) was transferred to a glass vial and then analyzed.

The LC-MS/MS analysis was performed using an Agilent 1260 Infinity (Agilent Technologies, Santa Clara, CA, USA), equipped with a degasser, autosampler, and binary pump coupled to a QTRAP 4000 hybrid triple quadrupole/linear ion trap mass spectrometer (AB Sciex, Framingham, MA, USA). Chromatographic separation was achieved with a Kinetex C-18 column (100 mm × 4.6 mm, particle size 2.6 µm) with a guard column supplied by Phenomenex (Torrance, CA, USA). The chromatographic and mass spectrometric conditions for *p*-cresol sulfate, *p*-cresol sulfate-d7, indoxyl sulfate, and indoxyl sulfate-d4 were applied according to our previous report [43].

### 5.3. Biochemical Parameters Measurements

All biochemical parameters were determined using the standard manufacturer’s procedures. Blood hemoglobin level was evaluated on XN-2000 (Sysmex Corporation, Kobe, Japan). Urinary proteins, hemoglobin, and glucose were quantified using the semi-quantitative automated reagent strip test Iris iRICELL2000 (Beckman Coulter, Brea, CA, USA). The dipstick urine test detects hemoglobin at four levels: 0.03, 0.10, 0.50, and ≥1.0 mg/dL; proteins at three levels: 10, 50, and 100 mg/dL; and glucose at three levels: 50, 100, and 200 mg/dL. Detection and quantification of the BK virus (BKV) and Cytomegalovirus (CMV) in the plasma were assessed with the real-time PCR method, using the diagnostic test R-gene Kit (bioMérieux, Marcy l’Etoile, France). The number of virus copies was automatically calculated by the ABI 7500 Fast Dx Real-Time PCR Instrument (Applied Biosystems, Foster City, CA, USA) relative to the standard curve. The creatinine concentration in serum was measured on a Cobas6000 (Roche, Risch-Rotkreuz, Switzerland). Next, eGFR was estimated from creatinine concentration, age, and sex of the subject using the chronic kidney disease epidemiology collaboration (CKD-EPI) equation according to the current 2012 Kidney Disease: Improving Global Outcomes (KDIGO) guidelines [18].

### 5.4. Statistical Analysis

Normally distributed data were expressed as mean and standard deviation, non-normally distributed data as median and interquartile range, and binary data as number and percentage. The statistical evaluation of the results was performed with STATISTICA software version 13.3 for Windows (TIBCO Software Inc., Palo Alto, CA, USA). To evaluate the normal distribution of the results, the Shapiro–Wilk test was performed. The patients’ characteristics at different time points were compared in a test for repeated variables, post hoc Dunn’s test with Bonferroni correction, and Tukey’s HSD test, as appropriate. Correlations were studied using the Spearman rank test. The logistic regression was used to build the model. Qualitative variables were coded as 0–1 dummy variables. Variables identified as statistically significant in the univariate analysis (*p* < 0.05) were included in the multivariate model. Variables were selected with backward elimination algorithms. To validate the model, 10-fold cross-validation was performed. Model calibration was estimated by the Hosmer–Lemeshow “goodness-of-fit” test [19]. The predictive accuracy of the model was assessed by calculating the area under the receiver operating characteristic (ROC) curve created from K-fold cross-validation [50]. Logistic regression was performed following the reporting guidelines for regression models [51]. Due to lack of significance, none of the interactions tested were included in a final model. A statistical evaluation of the model was also performed to avoid variable collinearity. Missing data were handled by case exclusion. The Kaplan–Meier method was used to estimate graft survival among populations stratified by predicted probability of the deterioration of graft function. The log-rank test was used to compare survival curves.

## Figures and Tables

**Table 1:** Clinical blood, saliva, and urine characteristics of the study population at M1, M6, and M12.

			M1 (*n* = 92)	M6 (*n* = 77)	M12 (*n* = 73)	*p*-Value		
			Frequency (%)/Median (IQR)			M1 vs. M6	M1 vs. M12	M6 vs. M12
gender, female/male			37/55 (40/60)			-		
age, years			53 (19)	55 (18)	56 (20)	-		
time post KTx, days			1125 (844)	1262 (889)	1476 (761)	-		
the drug, CyA/Tac/Eve			18/74/0 (20/80/-)	16/61/0 (21/79/-)	16/56/1 (22/77/1)	-		
smoking	current		13 (14)			-		
	ex-smoker		32 (35)			-		
	never		47 (51)			-		
	passive		16 (17)			-		
history of diabetes			18 (20)			-		
history of hypertension			55 (60)			-		
BMI, kg/m^2^			25.9 (4.4) ^1^	26.3 (4.6) ^1^	26.5 (4.6) ^1^	0.12347	0.00572	0.44694
serum creatinine, mg/dL			1.46 (1.03)	1.41 (0.85)	1.46 (0.88)	0.18631	0.76843	0.00807
eGFR, mL/min/1.73 m^2^			51 (48)	51 (49)	48 (45)	0.18631	0.76843	0.00807
CKD stage ^2^	1		9 (10)	9 (12)	6 (8)	-		
	2		29 (31)	22 (29)	24 (33)	-		
	3		30 (33)	27 (35)	25 (34)	-		
	4		21 (23)	15 (19)	13 (18)	-		
	5		3 (3)	4 (5)	5 (7)	-		
salivary IS, ng/mL			33 (37) ^3^	21 (39) ^4^	27 (39)	0.19572	0.79488	1.00000
serum IS, ng/mL			2075 (2788)	1715 (2489)	1870 (2497)	0.43288	1.00000	1.00000
salivary pCS, ng/mL			57 (139) ^3^	46 (86) ^4^	48 (78)	0.17769	1.00000	0.14575
serum pCS, ng/mL			7103 (9651)	4697 (6875)	4755 (6636)	0.36987	1.00000	0.50379
hemoglobin, g/L			136 (20) ^1^	136 (18) ^1,5^	134 (17) ^1,5^	0.89890	0.34903	0.16106
proteinuria ≥10 mg/dL ^12^			32 (35) ^5^	29 (32) ^6^	22 (24) ^7^	-	-	-
glucosuria ≥50 mg/dL			7 (7.7) ^5^	4 (5.3) ^6^	3 (3.3) ^7^	-	-	-
hematuria ≥0.03 mg/dL		UTI	25 (27) ^5^	17 (23) ^6^	17 (24) ^7^	-	-	-
		glomeruar	2 (2.2) ^5^	3 (4.0) ^6^	1 (1.4) ^7^			
BKV			3 (19) ^8^	1 (13) ^9^	1 (10) ^10^	-	-	-
CMV			1 (4) ^11^	0 (0) ^9^	0 (0) ^10^	-	-	-

BMI: body mass index; BKV: BK virus; CKD: chronic kidney disease; CMV: cytomegalovirus; CyA: cyclosporine; eGFR: estimated glomerular filtration rate (according to the CKD-EPI equation); Eve: everolimus; IS: indoxyl sulfate; KTx: kidney transplantation; pCS: *p*-cresol sulfate; Tac: tacrolimus; UTI: urinary tract infections; ^1^ mean (standard deviation); ^2^ according to KIDGO guidelines 2012 [18]; ^3^ *n* = 86, ^4^ *n* = 76, ^5^ *n* = 91, ^6^ *n* = 75, ^7^ *n* = 70, ^8^ *n* = 16, ^9^ *n* = 8, ^10^ *n* = 10, ^11^ *n* = 25, ^12^ results of semi-quantitative tests. The red color indicates statistical significance (*p* < 0.05).

**Table 2:** Correlations between salivary/serum IS and pCS and eGFR at M1.

r_s_ (*p*)	eGFR		
	All Subjects (*n* = 92)	DoGF-Free Group (*n* = 72)	DoGF Group (*n* = 20)
serum pCS	−0.50 (*p* = 0.00001)	−0.56 (*p* < 0.00001)	−0.52 (*p* = 0.01909)
salivary pCS	−0.55 (*p* < 0.00001) ^1^	−0.61 (*p* < 0.00001) ^2^	−0.47 (*p* = 0.06582) ^3^
serum IS	−0.78 (*p* < 0.00001)	−0.76 (*p* < 0.00001)	−0.71 (*p* = 0.00042)
salivary IS	−0.76 (*p* < 0.00001) ^1^	−0.75 (*p* < 0.00001) ^2^	−0.62 (*p* = 0.01024) ^3^

eGFR: Estimated glomerular filtration rate; IS: indoxyl sulfate; pCS: *p*-cresol sulfate; ^1^ *n* = 86, ^2^ *n* = 70, ^3^ *n* = 16.

**Table 3:** Salivary and urinary factors associated with the deterioration of graft function in kidney transplant in univariate and multivariate logistic analysis (*n* = 91).

	Univariate				Multivariate			
Variable at M1	β	OR	95% CI	*p*-Value	β	OR	95% CI	*p*-Value
salivary IS (per 10 ng/mL)	0.183	1.20	1.05–1.37	0.00682	0.170	1.19	1.04–1.35	0.01269
salivary pCS (per 10 ng/mL)	0.014	1.02	0.99–1.04	0.32124	–			
hematuria ^1^	0.936	2.55	0.92–7.06	0.07142	–			
proteinuria ^2^	1.485	4.42	1.56–12.52	0.00522	1.305	3.69	1.22–11.12	0.02048
glucosuria ^3^	1.806	6.09	0.94–39.36	0.05781	–			

IS: indoxyl sulfate; OR: odds ratio; pCS: *p*-cresol sulfate; ^1^ presence of urinary hemoglobin (0.03, 0.10, 0.50, ≥1.0 mg/dL) vs. negative result; ^2^ presence of urinary proteins (10, 50, 100 mg/dL) vs. negative result; ^3^ presence of urinary glucose (50, 100, 200 mg/dL) vs. negative result. The red color indicates statistical significance (*p* < 0.05).

## Data Availability

The data presented in this study are available on request from the corresponding author.
